# Systematic review of the relationship between family history and lung cancer risk

**DOI:** 10.1038/sj.bjc.6602769

**Published:** 2005-09-06

**Authors:** A Matakidou, T Eisen, R S Houlston

**Affiliations:** 1 Section of Cancer Genetics, Institute of Cancer Research, 15 Cotswold Road, Sutton, Surrey SM2 5NG, UK; 2 Section of Medicine, Institute of Cancer Research, Sutton, Surrey, UK; 3 Lung Unit, Section of Medicine, Royal Marsden Hospital, Downs Road, Sutton, UK

**Keywords:** family history, lung cancer, systematic review, meta-analysis

## Abstract

We performed a systematic review of 28 case–control, 17 cohort and seven twin studies of the relationship between family history and risk of lung cancer and a meta-analysis of risk estimates. Data from both case–control and cohort studies show a significantly increased lung cancer risk associated with having an affected relative. Risk appears to be greater in relatives of cases diagnosed at a young age and in those with multiple affected family members. Increased lung cancer risk was observed in association with an affected spouse and twin studies, while limited, favour shared environmental exposures. The limitations of the currently published epidemiological studies to infer genetic susceptibility are discussed.

Lung cancer as the most common cancer in the world represents a major public health problem (Parkin *et al*, 2005). Worldwide it accounts for approximately 1.2 million cancer-related deaths, while within the United Kingdom, there are approximately 33 600 deaths a year, the most common cause of cancer death in both men and women (Cancer Research UK, 2004a). Tobacco smoking is well established as the major aetiological risk factor for lung cancer, contributing to a 10-fold increase in risk in long-term smokers compared with nonsmokers (Doll and Peto, 1981; IARC, 2004). Other environmental risk factors include exposure to radiation, asbestos, heavy metals (arsenic, chromium, nickel), polycyclic aromatic hydrocarbons and chloromethyl ethers (IARC, 1986).

Lung cancer is frequently cited as an example of a malignancy solely attributable to environmental exposure. However, it has long been postulated that individuals may differ in their susceptibility to environmental risk factors. The only direct evidence for a genetic predisposition to date is provided by the increased risk of lung cancer associated with a number of rare Mendelian cancer syndromes as observed in carriers of constitutional TP53 (Hwang *et al*, 2003) and retinoblastoma (Sanders *et al*, 1989) gene mutations, as well as in individuals with xeroderma pigmentosum (Swift and Chase, 1979), Bloom's (Takemiya *et al*, 1987) and Werner's syndromes (Yamanaka *et al*, 1997).

Since the 1960s, various case–control and cohort studies of the relationship between family history and risk of lung cancer have provided some evidence of familial aggregation of lung cancer outside the context of the rare Mendelian syndromes. Here, we have systematically reviewed the published data on familial aggregation of lung cancer, with particular emphasis on the factors specific to lung cancer that influence our interpretation of the epidemiological evidence.

## MATERIALS AND METHODS

### Identification of studies

A search of the literature for articles that provided estimates of the familial risks of lung cancer was made using the electronic database PubMed (www.ncbi.nml.nih.gov/pubmed) for the years 1963 to May 2005. The search strategy included the keywords ‘lung cancer’, ‘risk’, ‘family history’ and ‘familial aggregation’. Studies were eligible if lung cancer risk was stratified by family history of lung cancer. All eligible studies were retrieved and bibliographies checked for other relevant publications. Review articles and bibliographies of other relevant studies were hand-searched to identify additional studies. Unpublished data were not sought.

Articles included for analyses were primary references and included case–control, cohort and twin studies. Care was taken to include only primary data or data which superseded earlier work. Details of the studies were extracted from published articles and summarised in a consistent manner to aid comparison.

### Statistical analysis

A meta-analysis was undertaken to obtain a pooled estimate of familial lung cancer risks from the published case–control and cohort studies. No distinction was made between studies that estimated familial risk from mortality or incidence data, respectively. For the purpose of this analysis, both the odds (OR) ratio and the ratio of observed to expected number of cases, were considered to represent relative risks (RR). Where both crude and adjusted estimates of risk were presented in studies, the adjusted estimates were used in the meta-analysis. The association between risk of lung cancer and family history of the disease was derived as a weighted average of study-specific estimates of the RR, using inverse variance weights (Kleinbaum *et al*, 1982). The logarithm of the RR (logRR) was assumed to have a normal distribution. If confidence intervals (CIs) were reported, standard errors (SEs) for the logRR were calculated. The logRR and the corresponding SEs were used as data points for the meta-analysis. In studies not quoting the RR or CIs, these were calculated from the presented data using two of the following parameters: the RR point estimate, the p-value, the O-E statistic (difference between numbers observed and expected) or its variance. Where no statistical parameters were presented the crude RR and its confidence intervals were calculated from the raw data.

Studies were analysed jointly using a random-effects model (DerSimonian and Laird, 1986), which takes into account heterogeneity among studies in addition to within-study variance. The percentage variability of the pooled RR attributable to heterogeneity between studies was quantified using the *I*^2^ statistic (Higgins and Thompson, 2002).

Meta-regression analysis was used to identify characteristics contributing to heterogeneity. The characteristics analysed included publication year (before or after 1993; the mean year of publication of studies), type of control group used, verification of the data collected, type of relative studied, sex of cases, adjustment for smoking habits in study subjects, adjustment for smoking habits in relatives and adjustment for family size. A random-effects weighted linear regression model was used, whereby the study-specific log RR was regressed on the study characteristic variable of interest (Thompson, 2001). The weights for the regression incorporated both the within-study variance as well as the between-study variance, estimated using maximum likelihood. Owing to the small number of studies in each meta-regression analysis, each study characteristic was examined in a univariate model. Results were expressed as a regression coefficient, which is the estimated increase or decrease in the logRR per unit increase in the covariate.

Evidence of publication bias was examined by generating Funnel plots of RRs (Egger *et al*, 1997). Studies are plotted in order of decreasing variance of the logRR. Horizontal lines represent 95% CIs. Each box represents the RR point estimate and its area is proportional to the weight of the study. The diamond (and broken line) represents the overall summary estimate, with CIs given by its width. The unbroken vertical line is at the null value (RR=1.0).

All statistical manipulations were undertaken using the program STATA version 8.0 (Stata Corporation, TX, USA) utilising the METAN and METAREG modules (Bradburn *et al*, 1999).

## RESULTS

### Case–control studies

In all, 31 studies were identified that provided risks of lung cancer stratified by family history of the disease (Tokuhata and Lilienfeld, 1963a, 1963b; Lynch *et al*, 1982; Ooi *et al*, 1986; Samet *et al*, 1986; Gao *et al*, 1987; Kramer *et al*, 1987; Sellers *et al*, 1987; Tsugane *et al*, 1987; Horwitz *et al*, 1988; Wu *et al*, 1988, 1996; McDuffie *et al*, 1989; Wu-Williams *et al*, 1990; Liu *et al*, 1991; McDuffie, 1991; Osann, 1991; Shaw *et al*, 1991; Pavlakou *et al*, 1993; Schwartz *et al*, 1996, 1999; Wang *et al*, 1996; Brownson *et al*, 1997; Kreuzer *et al*, 1998; Mayne *et al*, 1999; Bromen *et al*, 2000; Wunsch-Filho *et al*, 2002; Etzel *et al*, 2003; Wu *et al*, 2004; Jin *et al*, 2005; Matakidou *et al*, 2005). Three studies (Lynch *et al*, 1982; Sellers *et al*, 1987; Schwartz *et al*, 1999) were excluded from the review as the same data were duplicated in subsequent studies. Table 1 details the characteristics of the 28 eligible case–control studies. Sample sizes ranged from 85–2260 (median 563), with a total of 15 766 cases and 18 184 controls studied. The types of control subjects used varied between studies and included randomly selected community controls, hospital patients (with or without cancer) and spouses of cases. Data on the lung cancer status of relatives were collected in most studies via interview or questionnaire from the index case or a surrogate responder. Two studies used the medical records of participants to extract the relevant information, while five studies sought to verify information of the cancer status of relatives from death certificates or tumour registries.

**Figure 1:**
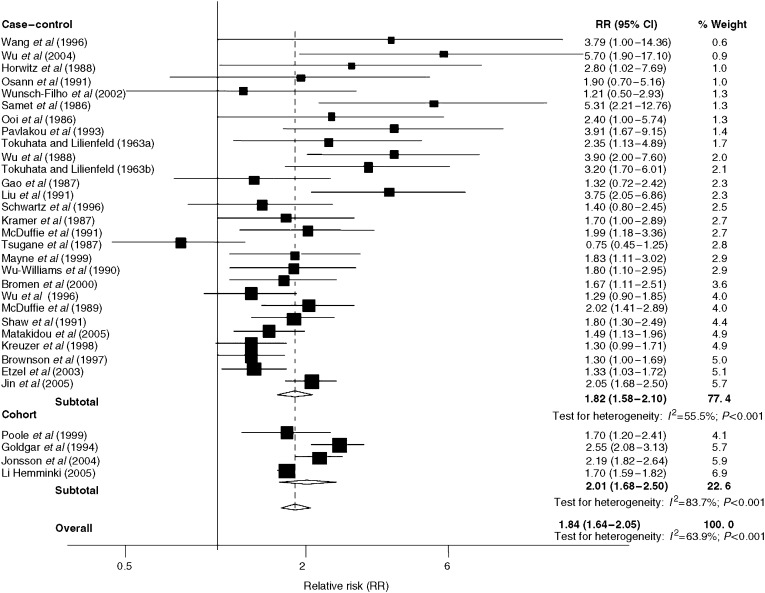
Forrest plot of Relative risks (RR) of lung cancer in the case–control and cohort studies examining the relationship between family history and lung cancer risk, CI=confidence interval.

Figure 1 shows a plot of the RRs of lung cancer associated with family history for all 28 case–control studies. The variables adjusted for in the analysis of each study are detailed in Table 1. In all, 27 of the studies demonstrated that family history of lung cancer was associated with an elevated risk in relatives. Of these, 21 were statistically significant. The pooled RR of lung cancer associated with having an affected relative from all 28 case–control studies was significantly elevated at 1.82 (95% CI: 1.58–2.10). There was evidence of significant heterogeneity between the contributing studies (*P*_het_<0.001; *I*^2^=59.0%).

Nine studies presented the RR of lung cancer associated with family history in subjects with an earlier age of onset of the disease. Three studies (Schwartz *et al*, 1996; Wu *et al*, 2004; Matakidou *et al*, 2005) selected the age of 60 years as the cutoff between younger and older subjects, (pooled RR 4.39; 95% CI: 1.33–14.42), three 55 years (Osann, 1991; Wu *et al*, 1996; Etzel *et al*, 2003) (pooled RR 1.10; 95% CI: 0.73–1.65), two 50 years (Tsugane *et al*, 1987; Bromen *et al*, 2000) (pooled RR 1.68; 95% CI: 0.28–10.12) and one study (Kreuzer *et al*, 1998) selected 45 years (RR 2.60; 95% CI: 1.10–6.15).

**Figure 2:**
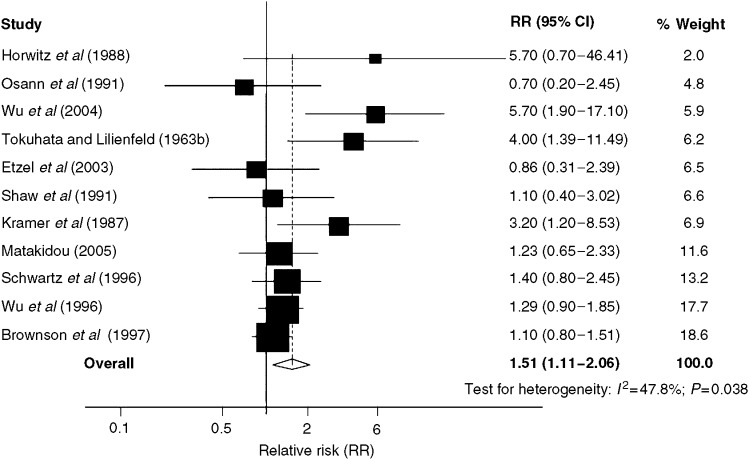
Forrest plot of familial lung cancer risks in never-smokers. RR=relative risks, CI= confidence interval.

In total 11 studies provided data specifically on never smokers (Figure 2). The pooled estimate of the RR across these studies was 1.51 (95% CI: 1.11–2.06). Six studies stratified lung cancer risks according to the number of affected relatives (Ooi *et al*, 1986; Shaw *et al*, 1991; Wu *et al*, 1996; Bromen *et al*, 2000; Jin *et al*, 2005; Matakidou *et al*, 2005). The pooled RR of lung cancer associated with a single affected relative was 1.57 (95% CI: 1.34–1.84) and for two or more affected relatives was 2.52 (95% CI: 1.72–3.70).

**Figure 3:**
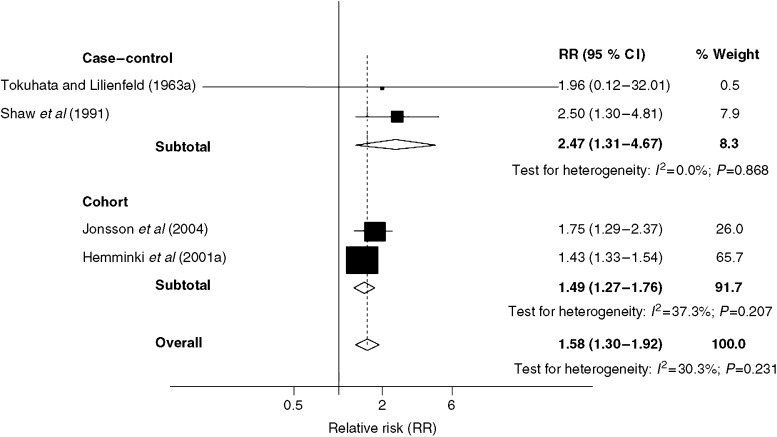
Forrest plot of Relative risks (RR) of lung cancer associated with history of lung cancer in a spouse. CI=confidence interval.

Figure 3 shows the RRs of lung cancer associated with history of lung cancer in the spouse of the participant, as estimated by two case–control studies. Pooling data from these studies, the RR was 2.47 (95% CI: 1.31–4.67).

### Cohort studies

Table 2 details the characteristics of the 17 cohort studies that have investigated the relationship between family history and lung cancer risk (Cannon-Albright *et al*, 1994; Goldgar *et al*, 1994; Hemminki *et al*, 1998, 1999; Hemminki and Vaittinen, 1999; Poole *et al*, 1999; Hemminki *et al*, 2001a, 2001b, 2004; Dong and Hemminki, 2001; Czene *et al*, 2002; Hemminki and Li, 2002, 2003; Li and Hemminki, 2003, 2004, 2005; Jonsson *et al*, 2004). In all, 13 of the studies (Cannon-Albright *et al*, 1994; Hemminki *et al*, 1998, 1999, 2001a, 2001b, 2004; Hemminki and Vaittinen, 1999; Dong and Hemminki, 2001; Czene *et al*, 2002; Hemminki and Li, 2002, 2003; Li and Hemminki, 2003, 2004) were excluded from the meta-analysis as their data were replicated in subsequent studies. From the studies examining the Swedish Family Cancer Database, the study by Li and Hemminki (2005) was included in the pooled analysis as it examined the largest data set.

Figure 1 shows the RRs of lung cancer associated with family history of the disease in the four cohort studies. All four studies demonstrated a significantly increased familial lung cancer risk. The pooled RR based on these studies was 2.01 (95% CI: 1.62–2.50). There was, however, evidence of significant heterogeneity between the studies (*P*_het_<0.001; *I*^2^=83.7%).

Young subgroups were presented in three studies, two of which (Jonsson *et al*, 2004; Li and Hemminki, 2005) defined these as those younger than 60 years (pooled OR 2.22; 95% CI: 1.08–4.57). The third study defined these as under the age of 64 years (RR of 2.53, 95% CI: 0.80–8.00; Goldgar *et al*, 1994).

Poole *et al* (1999) reported that probands with only one affected family member had an RR of 1.9 (95% CI: 1.3–2.7) of developing lung cancer, while the RR for those with two or more affected relatives or one affected relative below the age of 50 years was 1.1 (95% CI: 0.4–2.9). Dong and Hemminki (2001) reported an RR for probands with both a parent and sibling affected by lung cancer of 13.65 (95% CI: 2.57–40.41). RRs of lung cancer associated with lung cancer in the spouse (Figure 3) were reported by two studies, the overall RR being statistically increased (1.50; 95% CI: 1.27–1.76).

### Combined case–control and cohort studies

Pooling data from both the case–control and cohort studies (Figure 1), probands with a family history of lung cancer had an elevated risk of the disease, which was statistically significant, overall RR of 1.84 (95% CI: 1.64–2.05). Perhaps not surprisingly, there was evidence of heterogeneity across the studies (*P*_het_<0.001; *I*^2^=63.9%). Pooling data from the five studies estimating familial lung cancer risks for probands under the age of 60 years, the RR of lung cancer for this younger subgroup was 2.69 (95% CI: 1.58–4.58). Probands with a spouse affected by lung cancer (Figure 3) were also at an elevated risk of lung cancer (1.58, 95% CI: 1.30–1.92).

A meta-regression analysis was performed to investigate the contribution of study characteristics to the heterogeneity observed between the case–control (*n*=28) and combined (*n*=32) studies. Analysis was not performed for the cohort studies alone, as there were too few studies (*n*=4). The only variable significantly contributing to the heterogeneity observed was the year of publication of the studies analysed. Case–control studies published after 1993 reported lower RRs than studies published before this date (−0.31, 95% CI: −0.57, −0.04; *P*=0.02). Study design variables such as type of control group, sex of the study subject and type of relative examined did not significantly account for heterogeneity. Case–control and cohort studies that verified the family history data collected through death certificates or tumour registries reported higher RRs, although this did not reach statistical significance (0.22, 95% CI: −0.01, 0.45; *P*=0.06). Finally, variables pertaining to the type of RR adjustment applied by each study (smoking habits, family size) did not appear to affect the results of the meta-analysis. Studies adjusting for the smoking habits of the relatives reported lower RRs of lung cancer in association with family history, although not statistically significant (−0.24, 95% CI: −0.51, 0.04; *P*=0.09).

### Twin studies

Seven studies were identified (Harvald and Hauge, 1963; Braun *et al*, 1994; Braun *et al*, 1995a, 1995b; Ahlbom *et al*, 1997; Verkasalo *et al*, 1999; Lichtenstein *et al*, 2000) that have examined the lung cancer risk in cohorts of twins. Data in all studies have been collected either from death certificates or cancer registries. Four studies (Harvald and Hauge, 1963; Braun *et al*, 1995a; Ahlbom *et al*, 1997; Verkasalo *et al*, 1999) have been superseded by a later study (Lichtenstein *et al*, 2000) that combined data from three different national twin and cancer registries, while the study by Braun *et al* (1995b) replicates the data presented by the author's previous study (Braun *et al*, 1994). In total 121 424 twins have been examined for lung cancer concordance in two studies (Braun *et al*, 1994; Lichtenstein *et al*, 2000).

One study was based on a registry of almost 16 000 male twin pairs born between 1917 and 1927 who served in the armed forces in World War II, mortality being followed up from entry into the armed forces until the end of 1990 (Braun *et al*, 1994). The observed (O) frequency of twin pairs, both of which died of lung cancer, was compared with that expected (E) by chance. The O:E ratio among monozygotic twins (2.98; 95% CI: 1.55–5.56) did not exceed that of dizygotic twins (3.99; 95% CI: 2.35–5.79), the overall rate ratio being 0.75 (95% CI: 0.35–1.6). The study by Lichtenstein *et al* (2000) combined data on 44 788 pairs of twins listed in the Swedish, Danish and Finnish twin registries. Lung cancer concordance was estimated as the proportion of twin pairs with both twins affected of all ascertained twin pairs with at least one affected. For male twin pairs lung cancer concordance was 0.11 in monozygotic twins compared to 0.10 in dizygotic ones. For female twin pairs, lung cancer concordance was 0.09 and 0.01, respectively.

## DISCUSSION

The findings from our systematic review and meta-analysis of the published literature on familial aggregation of lung cancer are consistent with a two-fold increase associated with family history with evidence of risk being related to early age of diagnosis and number of relatives affected.

The interpretation of these studies requires caution: while familial risks are compatible with genetic predisposition, they could reflect common exposures. Smoking is the most important environmental risk factor of lung cancer, and the association between a person's smoking habits and that of his parents or siblings has been well documented (Salber and Macmahon, 1961). Unless adjustment is made for smoking habits, an above-expected incidence of lung cancer in relatives of lung cancer patients may be found, in the absence of any genetic effect. To date only four investigators (Table 1) have attempted to address this issue by taking into account the smoking habits of both the study subjects and their family members, reporting RRs comparable with those in studies making no such adjustment.

To minimise the impact of shared smoking habits in families, a number of studies have estimated familial risks associated with nonsmoker status (Figure 2). Pooling of the data in never-smokers resulted in an elevated risk of lung cancer associated with a family history of the disease that was statistically significant, supporting the view that genetic or other environmental factors may play a role in familial aggregations.

The contribution of shared environmental risk factors to familial lung cancer risk may also be assessed through risk estimation associated with an affected spouse since concordance of smoking habits between spouse pairs has been reported (Macken *et al*, 2000). Indeed, risk was significantly elevated in probands with an affected spouse, but remained lower than the risk associated with an affected relative, consistent with possible genetic factors.

Cohort studies of twins are classically used to separate genetic and environmental influences on familial aggregation of a disease. A critical assumption is that MZ and DZ twins display a comparable degree of similarity because of shared environmental factors, so that any difference in concordance rates only reflects genetic factors. The reported concordance ratios of lung cancer among male twins are almost equal, suggesting a strong environmental effect shared by twins (i.e. smoking behaviour) rather than a genetic component, which was widely cited to counter the propositions that an inherited basis exists for lung cancer or that the predisposition to smoke was itself genetic. Twin studies have, however, consistently shown greater concordance for smoking in MZ than DZ twins (Carmelli *et al*, 1992), suggesting that environmental exposure is being confounded by genetic influence. Yet, paradoxically, this concordance difference in smoking behaviour is not reflected in a concordance difference for lung cancer, although in female twins, where the prevalence is much lower, it did appear to follow a more conventional genetic pattern with risks in MZ being greater than in DZ twins, pointing to genetic predisposition (Lichtenstein *et al*, 2000).

One caveat to our meta-analysis is the significant heterogeneity observed between studies, although its impact on summary risk estimates is difficult to assess. Given the differences in location, design and control selection of the various studies, some degree of heterogeneity may be expected. Some of it is also likely to reflect differences in statistical methodology between studies, particularly in the adjustment for smoking habits. The presence or absence of adjustment for the smoking habits of study participants or their relatives did not appear to impact significantly on the results of our meta-analysis, although when adjustment was performed there was a trend towards reporting lower RR. A further issue inherent in many case–control studies is that of recall bias. The diagnosis of lung cancer in an individual may bring to light knowledge or awareness of lung cancer in relatives. Bias from this source can be eliminated by collecting the family history data before diagnosis (prospective/cohort study design). Alternatively, verification of cancer or cause of death among relatives from medical records or death certificates will eliminate recall bias. Where possible, we examined the impact of such verified data and noted that such studies reported higher rather than lower RRs; support that recall bias is unlikely to represent a significant confounder.

The only characteristic found to significantly impact on the heterogeneity observed between studies was the date of study publication. Studies published before 1993 reported higher RRs of lung cancer associated with positive family history, indicating time lag bias and possibly publication bias. However, formal testing showed no evidence of publication bias between case–control or cohort studies. Further statistical analysis of studies published before and after 1993 showed adjustment for family size to be a significant confounder. Individuals with large families are more likely to have an affected relative than those with small families; where average family size differs between cases and controls, failure to adjust for this might inflate the reported RR, as observed in the earlier studies. Univariate regression analysis of all the studies for the presence or absence of adjustment for family size did not, however, appear to account for the heterogeneity observed between studies, making it unlikely to significantly impact on the combined RR.

Type of control, type of relative studied and gender of participants were examined for their effect on the summary statistics with no significant associations detected. Although there were indications that some of these may have contributed to heterogeneity, each study possessed different combinations of both desirable and undesirable methodological features, such that no single factor, other than publication year, consistently increased or decreased RRs. Sample size limitations prevented detailed multivariate analysis, so that other important sources of heterogeneity may have become apparent if appropriate adjustment for confounding had been possible.

In summary, this systematic review finds a significant increase of lung cancer risk associated with having an affected relative, the risk being further increased with earlier age of onset of the disease and with multiple affected family members. This suggests that lung cancer risk may be in part genetically determined. However, familial studies of lung cancer are problematic as they display high heterogeneity and it is usually impossible to make a suitable adjustment for smoking, the major risk factor. Furthermore, the twin studies and the elevated lung cancer risk associated with an affected spouse do not favour a genetic susceptibility. Such limitations formally preclude the drawing of strong inferences about any genetic influences on lung cancer outside the context of rare Mendelian disorders. Ultimately, verification of a genetic predisposition must come from the identification of causal mutations. Recently, following a genomewide linkage scan, a candidate locus for lung cancer predisposition has been reported (Bailey-Wilson *et al*, 2004). If confirmed, this would provide the most convincing evidence to date of a genetic susceptibility outside rare Mendelian disorders.

## Figures and Tables

**Table 1:** Characteristics of case-control studies examining the relationship between family history and risk of lung cancer

			**Cases**	**Controls**						**Standardising variables**			
**Study**	**Region/country**	**Years of data collection**	**Number**	**Number**	**Type**	**Data sources** a	**Type of relative**	**Sex of cases**	**Recall bias**	**Age**	**Smoking^b^ (case/relative)**	**Family size**	**Other**
Tokuhata (1963b)	Maryland, USA	1960–61	270	270	Community	IS, NOK, OR, DC	1st degree	Both	Yes	Yes	Yes (qual)/yes (qual)	Yes	No
Tokuhata (1963a)	New York, USA	1957–60	361	722	Hospital (cancer free)	IS, NOK, DC	Parent/sibling	Both	Yes	Yes	No/no	No	Race, residence
Ooi (1986)	Louisiana, USA	1976–79	336	307	Spouse	IS, NOK, OR, DC	1st degree	Both	Yes	Yes	Yes (quant)/no	No	Occupation
Samet (1986)	New Mexico, USA	1980–82	518	769	Community	IS, NOK	Parent	Both	Yes	Yes	Yes (quant)/no	No	Ethnicity
Gao (1987)	Shanghai, China	1984–86	672	735	Community	IS	Parent	Female	Yes	Yes	Yes (quant)/no	No	Education
Kramer (1987)	New York, USA	N/A^c^	427	467	N/A	IS, NOK, TR	1st degree	Both	No	Yes	No/yes (qual)	No	Occupation
Tsugane (1987)	Tokyo, Japan	1976–85	185	185	Hospital (cancer free)	MR	1st/2nd degree	Both	Yes	Yes	No/no	No	Residence
Horwitz (1988)	New Haven, USA	1977–82	112	224	Hospital (nonsmoking-related cancers/cancer free)	MR	Parent/sibling	Female	Yes	Yes	No/no	No	Ethnicity
Wu (1988)	Los Angeles, USA	1983–86	336	336	Community	IS	Parent/sibling	Female	Yes	Yes	Yes (quant)/no	No	Ethnicity
McDuffie (1989)	Saskatchewan, Canada	1979–83	931	1031	Community	IS, NOK	1st degree	Both	Yes	Yes	No/no	No	Residence
Wu-Williams (1990)	Shenyang, China	1985–87	965	959	Community	IS	1st degree	Female	Yes	Yes	Yes (quant)/no	No	Education
Liu (1991)	Xuanwei, China	1985–86	110	426	Community	IS	1st/2nd degree	Both	Yes	Yes	Yes (quant)/no	No	Residence
Osann (1991)	California, USA	1969–77	208	208	Screening programme	IS	1st degree	Female	No	Yes	Yes (quant)/ no	No	Education, ethnicity
McDuffie (1991)	Saskatchewan, USA	1983–86	359	234	Community	IS, OR, MR, TR	Parent/sibling	Both	Yes	Yes	No/no	Yes	Residence
Shaw (1991)	Texas, USA	1976–80	937	955	Community	IS, NOK	1st degree	Both	Yes	Yes	Yes (quant)/no	Yes	Ethnicity, residence, ETS^d^
Pavlakou (1993)	Athens, Greece	1993	85	140	Community (cancer free)	IS	1st degree	Female	Yes	Yes	No/no	No	Ethnicity
Schwartz (1996)	Detroit, USA	1984–87	257	277	Community	IS, NOK, OR	1st degree	Both	Yes	Yes	N/a/no	No	Ethnicity, ETS, occupation
Wang (1996)	Guangdong, China	1990–93	390	390	Hospital (cancer free)	IS	N/A	Both	Yes	Yes	Yes (qual)/no	No	Residence, education
Wu (1996)	USA	1985–90	626	1240	Community	IS, NOK, OR	1st degree	Female	Yes	Yes	Yes (quant)/yes (qual)	Yes	Residence, ethnicity, education, ETS
Brownson (1997)	Missouri, USA	1986–91	618	1402	Community	IS, NOK, OR	1st degree	Female	Yes	Yes	No/yes (qual)	Yes	Ethnicity
Kreuzer (1998)	Germany	1990–96	2260	2319	Community	IS	Parent/sibling	Both	Yes	Yes	Yes (quant)/no	Yes	Residence, asbestos exposure
Mayne (1999)	New York, USA	1982–84	437	437	Community	IS, NOK, OR	1st degree	Both	Yes	Yes	No/no	Yes	Residence
Bromen (2000)	Bremen, Germany	1988–93	945	983	Community	IS	1st degree	Both	Yes	Yes	Yes (quant)/yes (qual)	Yes	Residence, ethnicity, asbestos exposure
Wunsch-Filho (2002)	Sao Paolo, Brazil	1989–91	285	578	Hospital (non-smoking related cancers/cancer free)	IS	1st degree	Both	Yes	Yes	Yes (quant)/no	No	Socioeconomic status
Etzel (2003)	Houston, USA	1995–00	806	663	Multidisciplinary clinic	IS	1st degree	Both	Yes	Yes	Yes (quant)/yes (qual)	No	Ethnicity
Wu (2004)	Taiwan	1992–02	108	108	Hospital (cancer free)	IS, NOK	1st degree	Female	Yes	Yes	No/no	No	Education, ETS, smoky coal exposure
Jin (2005)	Xuanwei, China	1992–99	740	740	Spouse	IS, NOK, OR, DC	1st degree	Both	Yes	Yes	Yes (quant)/no	Yes	Residence, smoky coal exposure
Matakidou (2005)	UK	1999–04	1482	1079	Spouse	IS	1st degree	Female	Yes	Yes	Yes (quant)/no	No	Ethnicity

a *Data sources*: IS=index subject; NOK=next of kin; OR=other relative; DC=death certificates; TR=tumour registry; MR=medical records.

b Qual=qualitative; quant=quantitative.

c N/A=not available.

d ETS=environmental tobacco smoke.

**Table 2:** Characteristics of cohort studies examining the relationship between family history and risk of lung cancer

							**Standardising variables**			
**Study**	**Region/country**	**Years of data collection**	**Number of cases**	**Data sources^a^**	**Type of relative**	**Sex of cases**	**Age**	**Smoking^b^** **(case/relative)**	**Family size**	**Other**
Cannon-Albright (1994)	Utah, USA	1952–92	2477	CR	Parent/sibling	Both	Yes	No/no	No	Sex, birthplace
Goldgar (1994)	Utah, USA	1952–92	2228	CR	1st degree	Both	No	No/no	No	No
Hemminki (1998)	Sweden	1958–94	N/A^c^	CR	Offspring	Both	Yes	No/no	No	Sex
Hemminki and Vaittinen (1999)	Sweden	1960–94	35 831	CR	Offspring	Both	Yes	No/no	Yes	No
Hemminki (1999)	Sweden	1958–94	N/A	CR	Parent	Both	Yes	No/no	N/A	No
Poole (1999)	USA	1959–72	877	IS	Parent/sibling	Female	Yes	Yes (qual)/no	Yes	Ethnicity, education, body mass index, hormonal factors
Hemminki (2001b)	Sweden	1958–94	N/A	CR	Parent/sibling	Both	No	No/no	No	No
Hemminki (2001a)	Sweden	1958–96	N/A	CR	Parent	Both	Yes	No/no	N/A	Sex
Dong (2001)	Sweden	1958–96	N/A	CR	Parent/sibling	Both	Yes	No/no	No	Sex
Hemminki (2002)	Sweden	1961–98	4524	CR	Parent	Both	Yes	No/no	N/A	Sex
Chene (2002)	Sweden	1958–96	N/A	CR	Parent/sibling	Both	No	No/no	No	No
Hemminki (2003)	Sweden	1961–98	N/A	CR	Parent	Both	Yes	No/no	N/A	Sex
Li (2003)	Sweden	1961–98	4524	CR	Parent	Both	Yes	No/no	N/A	Sex, region, period, socioeconomic status
Hemminki (2004)	Sweden	1991–00	5493	CR	Parent/sibling	Both	Yes	No/no	Yes	Sex, region, period, socioeconomic status
Li (2004)	Sweden	1991–00	5290	CR	Parent/sibling	Both	Yes	No/no	No	Sex, region, period, socioeconomic status
Jonsson (2004)	Iceland	1955–02	2756	CR	1st/2nd/3rd degree	Both	Yes	No/no	Yes	Sex
Li (2005)	Sweden	1961–00	55 238	CR	1st degree	Both	Yes	No/no	No	Sex, region, period, socioeconomic status

a *Data sources*: CR=cancer registry; IS=index subject.

b Qual=qualitative.

c N/A=not available.
